# Influence of *Hermetia illucens* Larvae Meal Dietary Inclusion on Growth Performance, Gut Histological Traits and Stress Parameters in *Sparus aurata*

**DOI:** 10.3390/ani13030339

**Published:** 2023-01-18

**Authors:** Ambra Rita Di Rosa, Letteria Caccamo, Lidia Pansera, Marianna Oteri, Biagina Chiofalo, Giulia Maricchiolo

**Affiliations:** 1 Department of Veterinary Sciences, University of Messina, Polo Annunziata, 98168 Messina, Italy; 2 Institute FOR MARINE Biological Resources and Biotechnology, National Research Council (IRBIM, CNR), Spianata S. Raineri 86, 98122 Messina, Italy

**Keywords:** black soldier fly, fishmeal substitution, gut histology, animal performance, gilthead seabream

## Abstract

**Simple Summary:**

The sustainability and further development of an intensive aquaculture of carnivores have been threatened in recent years by their dependence on fishmeal and fish oil. Alternative ingredients are therefore necessary to promote sustainable aquaculture production without compromising fish growth and health. Use of insects as an alternative protein source for aquaculture feed production is an excellent example of circular economy. Among insects, *Hermetia illucens* is one of the most promising sustainable protein and lipid sources. This study aimed to evaluate the effects of partially defatted HIM dietary inclusion on growth performance, stress indicators and gut histological traits of *Sparus aurata*. The feeding trial was carried out on 312 fish fed with a basal diet containing only fish meal as a protein source of animal origin, and three diets (HIM25, HIM35 and HIM50) containing 25%, 35% and 50% defatted *Hermetia illucens* meal as a partial replacement for fishmeal. The trial lasted 131 days. The results show that the insect meal inclusion did not affect growth performance and blood parameters and the health of the posterior gut tract, while the inclusion level at the 50% caused morphometric and histopathological changes in the anterior gut tract. Among the diets, the HIM35 was the most tolerated formulation by fish.

**Abstract:**

This study provided new data and knowledge on the potential use of *Hermetia illucens* meal (HIM) as a new sustainable ingredient for *Sparus aurata* diet. The effect of HIM dietary inclusion on fish growth performance, stress indicators and gut histology was studied. For 131 days, 312 fish were fed a basal diet containing fishmeal as animal protein source, and three diets containing 25%, 35% and 50% HIM as a partial replacement for fishmeal. The main findings indicated that fishmeal can be replaced by HIM up to 110 g/kg of substitution (35% of inclusion in diet) without negative effects on growth performance, stress parameters or histological traits of the posterior gut tract, and with positive effects (*p* < 0.05) on the histological and morphometric characteristics of the anterior gut tract. At the same time, the results showed that the effect of *Hermetia illucens* meal at 50% inclusion level caused morphometric and histopathological alterations in the anterior gut tract of seabream. In conclusion, this preliminary study suggested that the dietary inclusion level of HIM35 was the most tolerated by fish showing the best gut morphometric parameters and histological conditions, with fewer signs of inflammation, as well as good nutritional and health status.

## 1. Introduction

In recent years, the sustainability and growth of an intensive aquaculture of carnivores has been jeopardized by their reliance on fishmeal (FM) and fish oil (FO) [1]. To improve sustainable aquaculture without affecting fish performance and health and the quality of fillets, alternative ingredients are needed.

Many studies have focused on alternative protein sources, such as vegetable proteins (PP: oilseed flours, cereal proteins and grain legumes); however, carnivorous fish are not very efficient in digesting carbohydrates. Moreover, PP contains several anti-nutritional factors that reduce fish growth and morphological characteristics of the digestive system [2], decreasing the availability of nutrients [3]. Other alternative protein sources include processed animal proteins (PAPs) derived from animal by-products (poultry meal, blood meal and hydrolyzed poultry feathers) which provide essential amino acids [4], although this availability depends on the heat treatment adopted during feed manufacturing [5]. Among PAPs, insect meal is considered the optimal substitute for FM in fish diet [6,7,8,9]. An alternative protein source for the production of feed for aquaculture is represented by insects whose use is an excellent example of circular economy. In recent years, promising results in several commercial fish species have been obtained with the use of insects [10,11]. Insects are characterized by rapid growth and reproduction and are capable of bioconverting food waste or low-value organic substrates into high-quality nutrients [12]; they have a low environmental impact [13] and a high feed conversion ratio [14]. Hence, insect meals show many advantages over PAPs. Furthermore, the inclusion of insect meal in fish diet can modulate the gut microbiome [15,16,17] and stimulate the immune system [18] with positive repercussions on animal health [19].

The regulatory network on the use of insect-derived proteins in animal feeds is country-specific [20]. So far, in the EU, a list of PAPs from eight insects has been approved for aquaculture, poultry and pig feed [21].

Among these, *Tenebrio molitor* [22,23], *Musca domestica* [24,25] and *Hermetia illucens* [26,27,28,29] are the species that have received the most attention thanks to the possibility of mass breeding, the control of the life cycle and the chemical composition.

*Hermetia illucens* (HI) is one of the most promising sustainable protein and lipid sources [10,11,26,27,28,29,30,31]. The chemical composition of HI can vary considerably in relation to the breeding substrate and processing technology [32,33]. Based on dry matter (DM), the defatted HI meal contains 47.2% to 51.8% crude protein and 11.8% to 14.8% oil [34,35], while the full-fat HI meal contains 36% crude protein and 18% oil, on average [36]. The amino acid profile is comparable to that of fishmeal [9,37,38], while the fatty acid composition shows a deficiency of long-chain fatty acids (LCFA) of the n3 series such as eicosapentaenoic acid (EPA) and docosahexaenoic acid (DHA) [1]. However, studies report that the presence of fish offal and algae in the feeding media of the larvae can enrich the lipid profile in n3-LCFA [33,39]. Oteri et al. [28] report a similar content of alpha-linolenic acid, EPA and DHA among four diets for *Sparus aurata* containing increasing levels of HI flour replacing FM, confirming the hypothesis on the influence of the feeding substrate on the fatty acid composition of larvae [40]. A controversial issue with HI meal as an ingredient in the fish diet is the presence of chitin. Studies have shown that chitin may play a role in modulating gut microbiome [16] and innate immune response [41], although there are few studies on the effect of chitin on intestinal morphology, which is considered the main indicator of intestinal health [4,42,43].

The literature reported that HI meal could be an alternative solution to partially satisfy the nutritional needs of farmed fish without negatively affecting growth performance [39,42,44], condition factor, somatic indices, gut morphology and welfare [2,4,43,45].

In Italy and the Mediterranean area, gilthead seabream (*Sparus aurata*) and European sea bass (*Dicentrarchus labrax*) represent the dominant premium species of marine aquaculture. Although young, the production of seabream, which began in the late 1980s, continued to expand in the following decades. According to FEAP data, in 2019 seabream production in Europe was estimated at 199 thousand tonnes (a growing trend with respect to previous years), with a contribution from Italy of over 9 thousand tonnes [46].

To our knowledge, to date the use of HI meal as a partial replacement for fish meal in the diet of seabream has been performed by Karapanagiotidis et al. [44] on growth performance, feed utilization and body nutrient composition, and by Oteri et al. [29] and Pulido et al. [38] on the chemical and microbiological composition of fillets.

With the aim of further characterizing the feasibility of *Hermetia illucens* as an unconventional protein source in aquafeed closely related to the economic aspects of aquaculture farms, this trial focused on evaluating the possible influence of the inclusion of partially defatted HIM on growth performance, stress indicators and on the gut histology of *Sparus aurata*.

## 2. Materials and Methods

### 2.1. Ethics

All procedures on fish were carried out in accordance with Italian legislation [47] implementing the European Directive 2010/63/EU [48] on the protection of animals used for scientific purposes. The trial was carried out at the experimental aquaculture facility of IRBIM, CNR (Institute for Biological Resources and Marine Biotechnologies, National Research Council, Messina, Italy). The experimental protocol has received the authorization of the Italian Ministry of Health (Ministerial Authorization n. 491/2019-PR of the 9 July 2019).

### 2.2. Experimental Diets

Four diets, isoenergetic (approximately 22 MJ/kg, gross energy), isonitrogenous (approximately 43 g/100 g, as fed) and isolipidic (approximately 19 g/100 g, as fed), were formulated to meet the nutritional needs of *Sparus aurata* [49]. A basal diet (HIM0) was formulated with fishmeal (FM) as the exclusive protein source (250 g/kg) of animal origin. The other three diets (HIM25, HIM35 and HIM50), contained 25%, 35% and 50% *Hermetia illucens* defatted meal (as fed basis) as a partial replacement for fishmeal, corresponding to a different amount of FM (188, 163 and 125 g/kg) and HIM (79, 110 and 157 g/kg). In order to keep the diet isoenergetic, the quantities of the other ingredients utilized in the formulation were modified. In particular, the rapeseed oil was reduced from 100 g/kg (HIM0) to 98 g/kg in all the diet containing HIM, and wheat meal from 175 g/kg (HIM0) to 152 g/kg (HIM25), 142 g/kg (HIM35) and 129 g/kg (HIM50).

Diets were manufactured at SPAROS Lda (Olhao, Portugal) by extrusion in a pellet size of 4 mm; all the weighed, mixed and grounded dietary ingredients were extruded in a single screw extruder. Hence, after drying, the kibbles were coated with fish and rapeseed oils using vacuum coating technology. All diets were stored under vacuum at +4 °C until analyses. The ingredients and the nutritional characteristics of the diets (HIM0, HIM25, HIM35 and HIM50) are shown in Table 1. For the chemical composition, feed samples were analysed in duplicate for dry matter (DM), crude protein (CP), ether extract (EE), crude fiber (CF) and ash contents, according to AOAC [50] procedures (ID number: 2001.12, 978.04, 920.39, 978.10 and 930.05, respectively). The nitrogen-free extract (NFE, g/100 g, as fed) was calculated as:

NFE (g/100 g, as fed) = 100 − (crude protein + ether extract + crude fiber + ash).

The gross energy (GE, MJ/kg) content was determined using an adiabatic calorimetric bomb. As regards the composition of fatty acids, amino acids, minerals, the microbiological and sensory characteristics of the four diets, the analytical methods and the results obtained were reported in previous research [28].

### 2.3. Fish and Feeding Trial

On 3 February 2020, 312 *Sparus aurata* specimens, purchased at the Ittica Caldoli Company (Lesina, Foggia, Italy) were transported to the IRBIM-CNR facility (Messina, Italy) and transferred to a large tank (4.5 m^3^). Here, the fish were kept for approximately 1 month to acclimate to breeding conditions and fed with a commercial diet (46% protein, 16% fat, 20.7% NFE, 2.3% crude fiber; Aller Blue Omega 3 mm; Aller Aqua Company, Christiansfeld, Denmark). After the acclimation period, individual fish weights were determined (average initial weight: 143.65 ± 25.94 g); then, after a 24-h fasting period and light sedation (tricaine methanesulfonate solution, MS222, Sigma-Aldrich, Milan, Italy; 25 mg/L) to reduce the stress, the fish were randomly divided into twelve fiberglass tanks of 1.4 m^3^ (26 fish per tank) in an open circuit system equipped with a sand mechanical filter (filtering capacity: 4 micron; filtering speed: 30 m^3^/h/m^2^; filtered water flow: 12.7 m^2^/h) and UV lamp (15 m^3^/h; 40 mJ/cm^2^). During the experimental period, water flow was maintained constant (12 L/min–12 complete tanks renewals a day). Daily, a multi-parameter probe (YSI Professional Plus Multi-Parameters Water Quality Meter probe; Xylem Inc., Yellow Springs, OH, USA) was used to measure the water parameters: pH (range: 7.5–8.5), dissolved oxygen (range: 7.5–8.2 mg/L) and temperature (range: 14.4–26.3 °C). After subdivision in the 12 tanks, the fish for one week were gradually adapted to the experimental conditions and diets (HIM0, HIM25, HIM35 and HIM50); each diet was assigned in triplicate to the experimental groups (tanks) according to a completely random design (26 fish per tank, 3 replicated tanks per diet, 78 fish per diet).

Photoperiod followed natural changes according to the season of the year (February–August; latitude: 38°11′39′’48 N).

Six days a week for two daily meals (9:00 am and 4:00 pm) for 131 days, fish were fed by hand to visual satiety with the experimental diets (HIM0, HIM25, HIM35, HIM50), initially at 0.8%, increasing up to 1.5% of wet biomass according to water temperature. Any uneaten feed was removed 15 min after each meal by syphoning, dried overnight at 105 °C and weighed to estimate the feed intake.

Biomass tanks were weighed in bulk every 20 days throughout the growth experimental period to update the daily feeding rate.

Every day the tanks were inspected for fish mortality.

At the end of the trial, after a 24-h fasting period, to record some individual biometry measurements (total length and body weight) all fish were sacrificed through an overdose of anesthetic (tricaine methanesulfonate solution, MS222, Sigma-Aldrich, Milan, Italy; 0.3 g/L); in addition, liver, viscera and blood were sampled, as described in the following sections.

### 2.4. Growth Performance

On all fish (*n* = 78 fish per diet), the individual final body weight was recorded to calculate the following indices for each fish per each diet:

WG (Weight gain, g) = Final body weight (g) − Initial body weight (g)

$$\mathrm{SGR}\;\left(\mathrm{Specific}\;\mathrm{growth}\;\mathrm{rate},\;\%\right)=\left[\frac{\log\left(\mathrm{final}\;\mathrm{body}\;\mathrm{weight},\;g\right)-\log\left(\mathrm{initial}\;\mathrm{body}\;\mathrm{weight},\;g\right)}{\mathrm{number}\;\mathrm{of}\;\mathrm{feeding}\;\mathrm{days}}\right]\times 100\;\mathrm{FCR}\;\left(\mathrm{Feed}\;\mathrm{conversion}\;\mathrm{rate}\right)=\frac{\mathrm{total}\;\mathrm{feed}\;\mathrm{consumed}\;\mathrm{per}\;\tan k\;\mathrm{biomass}\;\left(g\;\mathrm{DM}\right)}{\mathrm{weight}\;\mathrm{gain}\;\left(g\right)}\;\mathrm{PER}\;\left(\mathrm{Protein}\;\mathrm{efficiency}\;\mathrm{ratio}\right)=\frac{\mathrm{weight}\;\mathrm{gain}\;\left(g\right)}{\mathrm{total}\;\mathrm{protein}\;\mathrm{fed}\;\left(g\;\mathrm{DM}\right)}\;\mathrm{DIR}\;\left(\mathrm{Daily}\;\mathrm{intake}\;\mathrm{rate},\;\%\right)=\left[\frac{\left(\mathrm{feed}\;\mathrm{intake}\;/\;\mathrm{mean}\;\mathrm{weight}\right)}{\mathrm{days}}\right]\times 100$$

### 2.5. Fulton’s Condition Factor and Somatic Indices

On all fish (*n* = 78 fish per diet), the individual total length was recorded to calculate the Fulton’s condition factor (K) for each fish per each diet:

$$K=\left[\frac{\mathrm{body}\;\mathrm{weight}\;\left(g\right)}{{\left(\mathrm{fish}\;\mathrm{total}\;\mathrm{length}\right)}^{3}\;\left(\mathrm{cm}\right)}\right]\times 100$$

From a subsample of 72 specimens (6 fish per tank, 3 replicate tanks per diet, 18 fish per diet), liver and viscera were separated and their weight was measured. Therefore, the hepato-somatic (HSI) and viscero-somatic (VSI) indices were calculated as follows:

$$\mathrm{HSI}\;\left(\%\right)=\left[\frac{\mathrm{liver}\;\mathrm{weight}\;\left(g\right)}{\mathrm{body}\;\mathrm{weight}\;\left(g\right)}\right]\times 100$$

### 2.6. Stress Parameters

On a subsample of 72 specimens (*n* = 18 fish per diet), blood samples (approximately 2 mL) were withdrawn from caudal veins to assay stress indicators. Blood samples were collected in specific serum separator tubes to obtain the sera or in VACUETTE tubes with heparin to separate plasma. For sera, blood samples were allowed to clot for two hours at 4 °C and then centrifuged for 15 min at 1000× *g*. For plasma, blood samples were centrifuged at 2500× *g* for 15 min following indications of protocol by Cusabio (Fish cortisol ELISA kit). Serum and plasma samples were divided in aliquots and stored at −80 °C until the analyses.

The serum adrenocorticotropic hormone (ACTH) and cortisol levels were determined by a microplate reader (iMark^TM^–Bio_Rad) using Enzyme Immunoassay kits supplied by CUSABIO^®®^ (Wuhan, China) (CSB-E15926Fh and CSB-E08487f, respectively). The serum glucose and lactate levels were determined by spectrophotometric analysis (Agilent Cary 60 UV-Vis, Shenyang, China) using commercial kits from SPINREACT (Glucose-LQ. GOD-POD liquid; Lactate. LO-POD. Enzymatic colorimetric). The plasma total protein concentration was determined using a PIERCETM BCA Protein Assay kit (Thermo Fisher Scientific, Beijing, China, Cat No: 23225).

### 2.7. Gut Histological Analyses

On a subsample of 72 specimens (6 fish per tank, 3 replicate tanks per diet, 18 fish per diet), the intestinal tract was excised, washed with a 0.9% saline solution to remove the content and quickly divided into two parts: anterior gut tract (AI), the tract following pyloric caeca, and posterior gut tract (PI), immediately pre- and post-ileorectal valve, including the rectum. From each segment, a 5 mm long piece was quickly cut and fixed in Bouin solution (Sigma-Aldrich, Italy) for 24 h and, afterward, washed and preserved in 70% ethanol until processing. Then, all the samples were dehydrated through a graded series of ethanol, clarified with xylene and embedded in paraffin wax blocks, sectioned at 5μm thickness using a rotatory microtome and stained with Haematoxylin & Eosin (H&E) and Periodic Acid Schiff for the identification of goblet cells.

A double-blinded histological examination to evaluate some possible pathological changes in the groups was performed using a light microscopy (Leica DMR) under the magnification of 5×, 10× and 40×. Morphometric parameters were determined by means of an images analyser (Leica Las V4.9).

A semi-quantitative analysis of the histopathological findings was performed using a scoring system modified [51,52,53] on a scale from 0–1 to 5 (score 0–1 indicates an intestine health status normal or compatible with the rearing condition, and score 5 is the most severe enteritis signs). In this scoring system, the following morphological parameters of enteritis were quantified independently:

1. Epithelium detachment from the lamina propria (ED);
2. Microvilli condition (mVC);
3. Loss of enterocytes nuclei position (NP);
4. Loss of normal supranuclear vacuolation (SNV).

The morphometric assessment of the gut segments was made on a total of 6 villi per sample, chosen in accordance with the literature [54,55]. Villi length (Vl) was also taken by measuring the length of the villi from the submucosa to the apex [54]. At last, the goblet cells (Gc) were quantified per villus, averaged for six villi, randomly selected in each section per fish and per dietary treatment [54].

### 2.8. Statistical Analysis

All data were checked for normal distribution and homogeneity of variances and normalized. Statistical analyses were carried out according to ANCOVA procedure of the XLSTAT statistical package [56]. For all the parameters, diet (HIM0, HIM25, HIM35 and HIM50) was used as a fixed effect. For the growth performances (SGR, FCR, PER, DIR), the initial body weight (IBW) was used as covariate. For the other data, the final body weight (FBW) was used as covariate. In this way, the possible effects of diet and body weight (IBW and FBW) have been separated. The results were expressed as means and pooled standard error of the mean (SEM). Comparison between means was performed by Tukey’s test, and differences were significant for *p* < 0.05.

## 3. Results

### 3.1. Growth Performance

During the trial, all diets were accepted by the fish and no mortality was observed.

The results on growth performance of gilthead seabream fed the experimental diets were reported in Table 2. Dietary inclusion of HIM did not significantly (*p* > 0.05) affect growth performance.

### 3.2. Somatic Indices and Fulton’s Condition Factor

The results of Fulton’s condition factor (K) are reported in Table 2 and those of the somatic indices (VSI and HSI) in Table 3.

The somatic indices and condition factor were significantly affected by the dietary treatments. Specifically, Fulton’s condition factor (K) showed a significantly (*p* < 0.01) lower value in fish fed the HIM35 diet than that in fish fed the basal diet (HIM0), while no significant (*p* > 0.05) differences were observed for the fish fed the HIM25 and HIM 50 diets compared to fish fed the HIM0 and HIM35 diets. The HSI showed a significantly (*p* < 0.05) lower value in fish fed the HIM35 diet than that in fish fed the HIM25 diet, while no significant (*p* > 0.05) differences were observed for the fish fed the basal diet (HIM0) and the diet with the highest inclusion of HIM (HIM50) compared to fish fed HIM25 and HIM35 diets. The VSI showed the significantly (*p* < 0.001) lowest value in the fish fed the HIM25 diet.

### 3.3. Stress Parameters

The results concerning stress parameters are reported in Table 4. No differences were highlighted for all the stress parameters between fish fed the experimental diets.

### 3.4. Gut Histological Investigations

**Figure 1:**
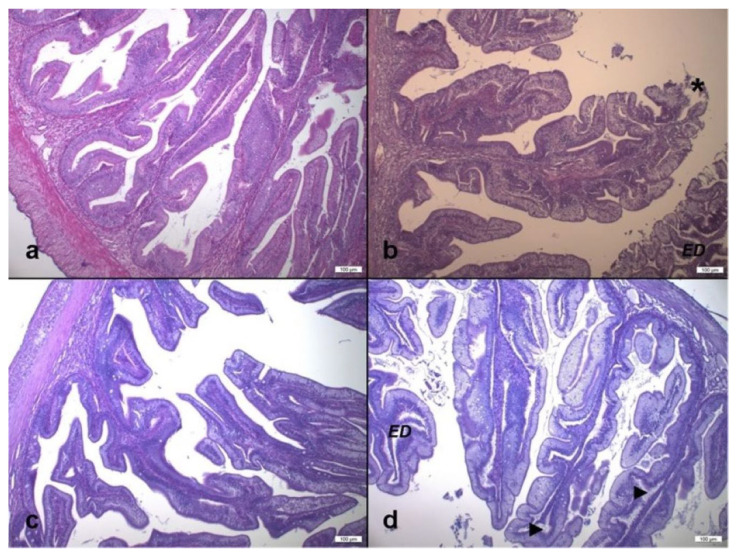
Transverse section of anterior intestine of *S. aurata*. (**a**) HIM0 group; (**b**) HIM25 group: subepithelial edema that determines the detachment of the epithelium from the lamina propria (**E**,**D**) and loss of cellular material (*) in the intestinal lumen, nuclei are strongly not aligned and supranuclear vacuolations (SNV) are altered; (**c**) HIM35 group: few and moderate gut morphology alterations, nuclei are largely aligned, SNV are moderately altered, few edema and no epithelium detachment. (**d**) HIM50 group: widening of lamina propria and impressive subepithelial edema (arrow head), strong alteration of nuclei position and SNV (H&E, magnification 10×).

The histological examination of the intestinal sections of all the experimental groups showed alterations with different levels of severity (Figure 1).

The main alterations observed consisted of morphological changes in the mucosa and submucosa layer structure, thickening of the lamina propria due to edema, detachment of the epithelium, misalignment of the enterocyte nuclei, alteration of the supranuclear vacuoles of the enterocytes and modification of the brush border.

In addition, leukocyte infiltrate, increased rodlet cells and flattened or atrophic villi in the posterior gut tract were observed describing a typical condition of enteritis.

In fish fed the HIM25 and HIM50 diets, more frequent and evident morphological changes were observed (Figure 1b,d). On the other hand, there were no substantial differences between HIM0 and HIM35 groups, where most fish showed few and moderate structural changes (Figure 1a,c).

#### 3.4.1. Gut Morphometric Investigations

Table 5 reports the effect of the dietary treatments on the morphometric parameters: villi length and villi width (six villi per sample) and goblet cells (per villus, averaged for six villi) of the anterior and posterior gut tracts. The villi length (Vl) of the anterior gut tract showed a significantly (*p* < 0.05) higher value in fish fed the HIM35 diet than that in fish fed the HIM25 diet. In the same gut segment, the villi width (Vw) showed significantly (*p* < 0.001) higher values in fish of the HIM35 and HIM50 groups than those of the HIM25 group; the goblet cells (Gc) showed significantly (*p* < 0.001) higher values in the fish of the HIM25 and HIM35 groups than those of the HIM0 and HIM50 groups. The villi width of the posterior gut tract showed a significantly (*p* < 0.05) higher value in the fish fed the HIM50 diet than those fed the basal diet (HIM0 group). No significant differences were reported for the villi length (*p* = 0.613) and goblet cells (*p* = 0.554) of the posterior gut tract.

#### 3.4.2. Gut Semi-Quantitative Analysis

Table 6 shows the results of the semi-quantitative scoring system of gut morphological changes on a scale from 0–1 to 5. The morphological changes concerned: epithelium detachment (ED); microvilli condition (mVC); nuclei position (NP); supranuclear vacuolation (SNV).

No significant differences were reported for ED, as well as for mVC of the anterior (*p* > 0.05) and posterior (*p* = 0.144; *p* = 0.122, respectively) gut tracts in relation to the dietary treatments. As regards NP and SNV, those of the posterior gut tract showed no significant differences between the dietary treatments (*p* > 0.05) while, in the anterior gut tract, NP and SNV showed significantly (*p* < 0.05) higher values in fish fed the highest inclusion of HIM (HIM50 group) than those in fish fed the basal diet (HIM0) and the diet with 35% of HIM inclusion.

## 4. Discussion

Dietary treatments containing 79 g/kg (HIM25), 110 g/kg (HIM35) and 157 g/kg (HIM50) of insect meal did not affect the gilthead seabream growth parameters (FCR, SGR, PER, DIR), indicating a similar nutrient bioavailability of diets containing HIM as compared to that containing FM, as also confirmed by the results of the proximal chemical composition reported by Oteri et al. [29] in a previous study that was part of the same research project.

These results are in agreement with several previous studies in which some levels of HIM inclusion (<50%) in seabream [44], rainbow trout [1], Atlantic salmon [42,57] and European sea bass [58] were tested. Nevertheless, results of defatted HIM inclusion in the seabream diet are still scarce and contradictory. This trial shows no significant differences between the groups for the final body weight; on the contrary, Karapanagiotidis et al. [44] observed a significant decrease in the final body weight in *Sparus aurata* fed diets containing HIM at 10%, 20% and 30% as a replacement for fish meal. The lack of unambiguous results in the studies carried out on HIM can be attributed to several reasons: level of HIM inclusion, fat level in the HIM used, presence of chitin, restricted vs. satiation feeding regime adopted, extrusion vs. pelleting process, and fish species and juvenile vs. adult fish stage of development [1]. Nairuti et al. [11], in a recent review on the use of HI larvae as a protein source for fish feeding, reported a possible substitution of up to 100% FM by HIM in diets for Jian carp and Nile tilapia and up to 75% FM by HIM in diets for African catfish, without negative effects on the growth performances. Furthermore, Nairuti et al. [11] observed that replacement levels of HIM, ranging from 10% to 50%, did not negatively affect growth parameters in most fish species, highlighting how, in some cases, the replacement can be positive. In Nile tilapia, the replacement of FM with HIM did not compromise growth performance and feed utilization efficiency indices, but also determined some positive effects on innate immunity and on parameters such as skin, mucus lysozyme and peroxidase activities [59]. 

Fulton’s condition factor (K) provides information on the condition, fatness, or wellbeing of fish. When K values are lower than 1 the fish are not in a good state of well-being within their habitat, while values higher than 1 indicate that the fish are in a good physiological state of well-being [60].

In this trial, K values results were higher than 1 independently from the diet, reflecting a good state of well-being, and they were significantly affected by the diet, reflecting a change in fish fat deposition. The significant differences observed for VSI in fish fed diets containing HIM at 25% and 35% of inclusion compared to the FM-based diet are due to the mesenteric fat, which is the main storage site, they are not related to liver weight [61]. In fact, HSI showed similar values between fish fed diets containing HIM compared to those fed the basal diet (HIM0), confirming the absence of problems related to the ingestion of HI compared to FM. One explanation could be related to the fatty acid composition of insect meal which could alter hepatic lipid accumulation [62]. Studies performed on zebrafish [63,64,65,66] reported that the n6/n3 ratio seems to be the key factor in determining hepatic lipid deposition, providing information on the effects and suitability of HIM-based diets [1,67,68]. In this trial, the fatty acid composition as well as the n6/n3 polyunsaturated fatty acid ratios (n6/n3 PUFA, HIM0: 1.16; HIM25: 1.14; HIM35: 1.14; HIM50: 1.12) of all diets showed similar results [28].

Some hematological parameters such as cortisol, glucose, lactate and proteins can be used to evaluate fish health and/or stress conditions [69,70,71]. Studies report that stressed conditions increase the blood levels of cortisol [72,73,74,75] and glucose [76,77,78], and many researchers believe that stressed fish “as a rule” exhibit high blood levels of cortisol and glucose [79]. Furthermore, a correlation between diet and blood indicators such as cortisol, glycemia, lactate, proteins and ACTH is known [80,81]. In this study, the effect of HIM dietary inclusion on seabream health condition was evaluated measuring primary and secondary stress response parameters (ACTH, cortisol, glucose, lactate and total proteins). The results showed that none of the blood parameters differed significantly in relation to the dietary treatments, confirming that HIM did not affect the health condition of the seabream, as also confirmed by Fulton’s condition factor at greater than 1 in all experimental fish. These findings are consistent with the observations of Zhou et al. [82] in Jian carp fed diets containing different levels of HIM (35, 70, 105 and 140 g/kg) as replacement of FM and with those of Yildirim-Aksoy et al. [83] in hybrid tilapia (*O. niloticus × O. mozambique*) specimens fed a diet containing HIM diet (30% FM replacement) for 12 weeks.

Histopathological analysis of the intestine is considered one the main approaches to evaluate fish health and nutritional status [84]. Since insect meal is known to include different molecules such as chitin and short-medium fatty acids, which may have an important role in gut welfare regulation [16,85,86], the analysis of histopathological indices has recently been applied to several studies in order to provide information on possible inflammation and/or alterations in the nutrient transport in fish [87].

Among these indices, the intestinal morphology is considered one of the main indicators of fish health since its morphological structure rapidly and often reversibly changes in response to dietary inputs [88]. The literature reports that the use of insect meal as an ingredient in fish diet could affect intestinal morphology by modifying its structure by means of changes in mucosa thickness [43], in villi morphometry and goblet cells numbers [89] and, also, by increasing inflammatory cells [90].

The length of intestinal villi is one of the main parameters studied in the feeding trials with alternative protein sources even if the results obtained often appear contradictory. Some authors reported a decrease in the length of villi [1,62,88,91,92,93], while others have highlighted how the length of the villi increases or does not change in the anterior or posterior gut tracts, following the inclusion of HIM into the diet [43,88,94]. In this trial, the villous length of the anterior tract of the intestine was greater in fish in the HIM35 group than in those in the HIM25 group, and, however, in all the groups it was comparable to that of fish fed the basal diet (HIM0). Since proteins are mainly digested in the anterior gut tract and to a lesser extent in the hind tract [1], this could explain why the dietary treatments containing proteins from insect meal did not affect the gilthead seabream growth parameters.

Another criterion usually used to establish the state of health of the intestinal mucosa is the evaluation of the thickness of the villi and, also in this case, the literature reports conflicting results.

In this study, the replacing of FM with 50% of HI meal (HIM50) in the posterior gut tract of gilthead seabream determined a significant increase in villi width. This could be partly due to the presence of subepithelial edema, which is one of the signs of inflammation that we have found, especially in the HIM50 group. Conversely, Melenchon et al. [91] in rainbow trout fed with a diet containing 50% of HI meal as a replacement of FM, and Fronte et al. [95] in zebrafish fed diets including increasing levels of full-fat HI meal (with respect to FM), did not observe any changes. Recently, Li et al. [87] reported that a partial (40%) up a total substitution of partially defatted HI meal did not cause negative effects on gut morphology in Atlantic salmon, indicating a high tolerance of salmonids to high dietary HIM inclusion levels.

In the present study, the goblet cells were studied along the intestinal tract since they are very important for fish nutrition and health. The mucus secreted by the goblet cells is involved in the epithelial protection as well as in the lubrication for the passage of nutrients; it supports waste exclusion [96] by lubricating undigested material for progression into the rectum and it plays an active role in the intestinal immune response of fish [97]. In this trial, an increasing amount of goblet cells were observed in the anterior gut tract of fish fed the diets containing 25% and 35% of HIM inclusion while, in the posterior gut tract, similar values of goblet cell abundance between the diets were observed. In this regard, contradictory results are reported in the literature. Cardinaletti et al. [1] observed an increasing amount of mucin goblet cells in the gut tract of rainbow trout fed HIM inclusion. Similar results were obtained by Randazzo et al. [45,98] in rainbow trout fed diets in which 30% or 60% of vegetable proteins were replaced with defatted HIM. On the contrary, Elia et al. [2] did not observe significant differences in mucous cells in rainbow trout, independently of partially defatted HIM dietary inclusion (25 or 50%). Recently, studies on the administration of HIM-based diets in zebrafish were carried out. A general increase in the number of mucous cells was observed when zebrafish were fed exclusively on full-fat HIM [63], while no changes in zebrafish fed diets containing an inclusion of HIM in partial substitution of FM were observed [65,66,99].

With the aim of ascertaining any inflammatory states, a semi-quantitative evaluation of the anterior and posterior gut tracts based on epithelial detachment, microvilliosal condition, position of the nuclei and supranuclear vacuolization was performed.

Along the intestinal tract, no significant differences in the parameters of mucosa epithelium detachment (ED) and microvilli conditions (MVc) were observed. As regards the nuclei position and supranuclear vacuoles, significant changes in the anterior gut tract were observed. In particular, the highest value (score 4) of both nuclei position and supranuclear vacuoles was found in fish fed the highest level of HIM (HIM50) indicating severe signs of enteritis and an inflammatory condition in the anterior gut tract of the fish belonging to the HIM50 group. This result could be due to the high chitin content of the HIM50 diet and to the low number of goblet cells in the anterior gut tract of the HIM50 group, which induced a less intestinal tract lubrification for the passage of dietary ingredients [96]. Similar intestinal pathological changes induced by high levels of HI meal into the diet were observed in Siberian sturgeon [43], Mirror carp [100] and Jian carp [101].

## 5. Conclusions

This study provided new data and knowledge on the potential use of *Hermetia illucens* meal as a new sustainable ingredient for carnivorous fish diets. The main findings indicated that fishmeal can be replaced by *Hermetia illucens* meal up to 110 g/kg of substitution (35% of inclusion in diet) in a practical diet for *Sparus aurata* without negative effects on growth performance, stress parameters and histological traits of the posterior gut tract and with positive effects on the histological and morphometric characteristics of the anterior gut tract. At the same time, the results showed that the effect of *Hermetia illucens* meal at 50% inclusion level (157 g/kg of substitution) caused morphometric and histopathological alterations in the anterior gut tract of seabream, although it did not modify growth performance and stress parameters. 

Overall, this preliminary study suggested that the dietary inclusion level of HIM35 was the most tolerated by fish showing the best gut morphometric parameters and histological conditions, with fewer signs of inflammation, as well as good nutritional and health status.

Further investigations on digestibility and gut microbiota composition are being carried out to better understand the suitability of *Hermetia illucens* as a dietary ingredient in farmed fish and its economic feasibility compared to currently used fishmeal.

## Figures and Tables

**Table 1:** Diet ingredients and proximate composition of the experimental diets.

	HIM0	HIM25	HIM35	HIM50
Ingredients, % as fed				
Fish meal	25.00	18.75	16.25	12.50
*Hermetia illucens* meal	0	7.90	11.00	15.70
Soy protein concentrate	5.00	5.00	5.00	5.00
Wheat gluten	5.00	5.00	5.00	5.00
Corn gluten	5.00	5.00	5.00	5.00
Soybean meal 48	15.00	15.00	15.00	15.00
Rapeseed meal	5.00	5.00	5.00	5.00
Wheat meal	17.45	15.17	14.21	12.88
Whole peas	4.00	4.00	4.00	4.00
Fish oil	5.00	5.00	5.00	5.00
Rapeseed oil	10.00	9.80	9.80	9.80
Vitamin and mineral premix	1.00	1.00	1.00	1.00
Vitamin C35	0.03	0.03	0.03	0.03
Vitamin E50	0.02	0.02	0.02	0.02
Antioxidant	0.30	0.30	0.30	0.30
Sodium propionate	0.10	0.10	0.10	0.10
MCP, monocalcium phosphate	1.50	2.20	2.50	2.80
L-Lysine	0.30	0.35	0.37	0.40
L-Tryptophan	-	0.03	0.04	0.05
DL-Methionine	0.10	0.15	0.18	0.22
L-Taurine	0.20	0.20	0.20	0.20
Chemical composition, % as fed				
Dry matter	92.33	92.78	92.90	92.64
Crude protein	42.7	42.7	42.7	42.7
Ether extract	18.6	18.6	18.6	18.7
Crude fiber	2.3	2.2	2.2	2.1
Ash	9.3	9.3	9.4	9.3
NFE *	19.43	19.98	20.00	19.84
Gross Energy, MJ/kg feed ^#^	22.0	21.9	21.9	21.9

HIM0 = control; HIM25 = 25% replacement level of FM with HIM; HIM35 = 35% replacement level of FM with HIM; HIM50 = 50% replacement level of FM with HIM; * Nitrogen-free extract, NFE (%) = 100 − (%Crude Protein + %Ether extract + %Crude fiber + %Ash). ^#^ Determined by calorimetric bomb.

**Table 2:** Growth performance of gilthead seabream fed the experimental diets.

	GROUP				*p-*Value		SEM
	HIM0	HIM25	HIM35	HIM50	D	IBW	
Number of fish	78	78	78	78			
IBW, g	144	144	144	144			2.883
FBW, g	387	386	396	394	0.473	0.762	5.516
FCR	1.42	1.43	1.41	1.42	0.979	0.866	0.030
SGR, %/d	0.76	0.76	0.76	0.74	0.774	0.758	0.014
PER	1.82	1.80	1.77	1.76	0.746	0.829	0.041
DIR, %	18.30	18.43	17.77	17.56	0.137	0.261	0.269
K	1.908 ^a^	1.867 ^ab^	1.828 ^b^	1.852 ^ab^	0.008	<0.0001	0.016

HIM0 = control; HIM25 = 25% replacement level of FM with HIM; HIM35 = 35% replacement level of FM with HIM; HIM50 = 50% replacement level of FM with HIM; D = Diet; IBW = Initial Body Weight; FBW = Final Body Weight; FCR = Feed Conversion Ratio; SGR = Specific Growth Rate; PER = Protein Efficiency Ratio; DIR = Daily Intake Rate; K = Fulton’s Condition Factor; SEM = Standard Error of the Mean. Mean values with different letters within the same row are significantly different for *p* < 0.05.

**Table 3:** Somatic indices of gilthead seabream fed the experimental diets.

	GROUP				*p*-Value		SEM
	HIM0	HIM25	HIM35	HIM50	D	FBW	
Number of fish	18	18	18	18			
Body weight, g	394	407	406	410	0.114		5.163
VSI, %	0.251 ^a^	0.212 ^c^	0.234 ^b^	0.239 ^ab^	<0.0001	0.544	0.004
HSI, %	0.065 ^ab^	0.104 ^a^	0.062 ^b^	0.090 ^ab^	0.018	<0.0001	0.011

HIM0 = control; HIM25 = 25% replacement level of FM with HIM; HIM35 = 35% replacement level of FM with HIM; HIM50 = 50% replacement level of FM with HIM; D = Diet; FBW = Final Body Weight; HSI = Hepato-somatic Index; VSI = Viscero-somatic Index; SEM = Standard Error of the Mean. Mean values with different letters within the same row are significantly different for *p* < 0.05.

**Table 4:** Stress parameters of gilthead seabream fed the experimental diets.

	GROUP				*p*-Value		SEM
	HIM0	HIM25	HIM35	HIM50	D	FBW	
Number of fish	18	18	18	18			
Cortisol, ng/mL	21.693	19.972	19.166	18.492	0.541	0.261	1.623
Glucose, mg/DL	63.371	66.715	63.277	65.317	0.799	0.754	2.829
Lactate, mg/DL	19.010	20.555	19.082	18.782	0.867	0.323	1.631
Total Proteins, mg/mL	33.495	33.197	30.134	31.118	0.448	0.936	1.693
ACTH, pg/mL	141.272	145.767	147.839	146.571	0.991	0.997	14.942

HIM0 = control; HIM25 = 25% replacement level of FM with HIM; HIM35 = 35% replacement level of FM with HIM; HIM50 = 50% replacement level of FM with HIM; D = Diet; FBW = Final Body Weight; ACTH = adrenocorticotropic hormone; SEM = Standard Error of the Mean. Mean values with different letters within the same row are significantly different for *p* < 0.05.

**Table 5:** Intestinal morphometric parameters of gilthead seabream fed experimental diets.

	GROUP				*p*-Value		SEM
	HIM0	HIM25	HIM35	HIM50	D	FBW	
Number of fish	18	18	18	18			
Vl Anterior gut, μm	1593.798 ^ab^	1430.240 ^b^	1638.236 ^a^	1487.868 ^ab^	0.024	0.110	53.511
Vl Posterior gut, μm	1106.586	1107.966	1086.043	1174.447	0.613	0.022	49.475
Vw Anterior gut, μm	306.016 ^ab^	287.608 ^b^	341.124 ^a^	352.785 ^a^	0.002	0.939	13.237
Vw Posterior gut, μm	153.452 ^b^	159.187 ^ab^	165.904 ^ab^	177.309 ^a^	0.033	0.513	5.945
Gc Anterior gut, nr.	139.722 ^b^	173.763 ^a^	171.066 ^a^	134.569 ^b^	<0.0001	0.321	6.906
Gc Posterior gut, nr.	128.119	135.355	124.173	119.603	0.554	0.053	7.98

HIM0 = control; HIM25 = 25% replacement level of FM with HIM; HIM35 = 35% replacement level of FM with HIM; HIM50 = 50% replacement level of FM with HIM; D = Diet; FBW = Final Body Weight; Vl = Villi length; Vw = Villi width; Gc = Goblet cells; SEM = Standard Error of the Mean. Mean value with different letters within the same row are significantly different for *p* < 0.05.

**Table 6:** Semi-quantitative scoring analysis (score 0–5) of morphological changes of the intestine of gilthead seabream fed the experimental diets.

	GROUP				*p*-Value		SEM
	HIM0	HIM25	HIM35	HIM50	D	FBW	
Number of fish	18	18	18	18			
ED Anterior gut	3.495	3.041	3.216	3.582	0.459	0.059	0.267
ED Posterior gut	2.323	3.098	3.050	3.363	0.144	0.164	0.325
mVC Anterior gut	1.737	2.051	1.665	2.213	0.444	0.571	0.272
mVC Posterior gut	1.521	2.510	2.060	2.020	0.122	0.219	0.285
NP Anterior gut	2.963 ^b^	3.772 ^ab^	3.220 ^b^	4.433 ^a^	0.002	0.491	0.278
NP Posterior gut	3.086	3.047	2.996	2.927	0.987	0.334	0.323
SNV Anterior gut	2.670 ^b^	3.260 ^ab^	2.659 ^b^	3.966 ^a^	0.004	0.036	0.282
SNV Posterior gut	2.408	3.050	2.886	3.433	0.199	0.557	0.335

HIM0 = control; HIM25 = 25% replacement level of FM with HIM; HIM35 = 35% replacement level of FM with HIM; HIM50 = 50% replacement level of FM with HIM; D = Diet; FBW = Final Body Weight; SEM = Standard Error of the Mean. ED = Epithelium Detachment; mVC = microvilli condition; NP = Nuclei Position; SNV = Supranuclear Vacuoles; ant = anterior gut; post = posterior gut. Mean value with different letters within the same row are significantly different for *p* < 0.05.

## Data Availability

Not applicable.
